# Tympanic membrane temperature in adopted children associated with sleep problems and pre-adoption living arrangements: an exploratory study

**DOI:** 10.1186/s40359-014-0051-2

**Published:** 2014-12-03

**Authors:** Rani C Damsteegt, Marinus H van IJzendoorn, Dorothée Out, Marian J Bakermans-Kranenburg

**Affiliations:** Centre for Child and Family Studies, Leiden University, Leiden, The Netherlands

**Keywords:** Tympanic membrane temperature, Sleep problems, Behavior problems, Adoption

## Abstract

**Background:**

Tympanic membrane temperature (TMT) has been proposed as an indicator of cerebral activation and TMT asymmetry may indicate lateralization, which has been associated with specific (problem) behaviors in children and adults. The current study explored the relations between pre-adoption living arrangements, TMT, and behavior and sleep problems in a sample of adopted toddlers.

**Methods:**

Ninety-two families who had adopted a Chinese girl who had previously been placed in an institution or foster care reported on behavior problems using the Child Behavior Checklist and TMT two months (Time 1) and six months (Time 2) after adoption.

**Results:**

Structural equation modeling revealed that institutionalized children had significantly higher left than right TMTs compared with foster care children at Time 2. A higher left than right TMT was associated with increased sleep problems and total behavior problems at Time 1, but not at Time 2.

**Conclusions:**

Our findings with regard to pre-adoption living arrangements, TMT asymmetry, and sleep problems suggest that TMT is sensitive to early environmental influences and may be a biological marker of vulnerability to the development of sleep problems in children from adverse backgrounds.

## Background

Tympanic membrane temperature (TMT) or ear temperature may not only be an indicator of physical well-being, but has also been proposed as an indicator of cerebral temperature and hemispheric lateralization (Schiffer et al. 1999; Cherbuin and Brinkman 2004; Propper and Brunyé 2013). Because it is a non-invasive method that requires minimal resources, it may be an attractive method to measure neural activity in addition to other methods (Boyce et al. 2002; Propper et al. 2013). Furthermore, as (unilateral and bilateral) TMT has been associated with various behavior problems (Boyce et al. 1996; Propper and Brunyé 2013), it may be a relevant measure for individual differences in behavioral associations. Since adopted children are at risk for developing behavior problems (Hawk and McCall 2010; Juffer and Van IJzendoorn 2005) and come from diverse and sometimes adverse backgrounds, we explored relations between pre-adoption experiences, TMT, and behavior problems in adopted children.

TMT is assumed to reflect cerebral temperature and hemispheric activity (Schiffer et al. 1999; Cherbuin and Brinkman 2004; Gunnar and Donzella 2004). Schiffer et al. (1999) found that lateral visual field stimulation was related to both EEG and TMT laterality and conclude that TMT asymmetry could reflect changes in cerebral blood flow. Findings of associations between specific behaviors and motivations such as activation versus inhibition (Helton 2010) and frontal EEG asymmetry illustrate the importance of cerebral asymmetry (Boyce et al. 2002). In line with this proposition, several studies have found associations between TMT asymmetry and behavior in children and adults (Propper and Brunyé 2013). However, the mechanisms underlying the relation between TMT, brain activity, and behavior have been under debate: does a higher temperature on one side indicate ipsilateral activation increase or decrease (Propper et al. 2013)? Furthermore, whether one views TMT as a stable or dynamic trait determines the design of the study: between-subject designs relate cerebral lateralization to trait differences, whereas within-subject designs compare a subject’s baseline measure with lateralized responses to a certain task (Helton 2010; Propper et al. 2013). Consequently, results from between-subject designs may indicate whether individual differences in baselines are present, whereas results from studies using within-subject designs may provide information on individual differences in reactivity, in response to specific tasks or situations (e.g., Cherbuin and Brinkman 2004; Jones et al. 2011).

In addition, findings from previous studies have been inconsistent in concluding whether a higher right than left-sided TMT is associated with more negative or positive behaviors (Boyce et al. 2002; Propper and Brunyé 2013). For example, Boyce et al. (1996) found that higher left TMT (higher left temperature as compared to right ear temperature) was associated with various internalizing and externalizing problems such as aggression, schizoid behaviors, social withdrawal, depression, and somatic behavior symptoms in 8-year-old children. A higher right-sided temperature was associated with more ego resilience. In contrast, six years later Boyce et al. (2002) found that 4- to 8-year-old children with higher right-sided temperatures displayed more behavior problems, whereas more positive and socially competent behaviors were shown by children with higher left-sided temperatures. Findings of Gunnar and Donzella (2004) were consistent with these results: higher right than left temperatures were associated with sadness, and higher left temperatures were associated with increased laughing and smiling in 3- to 5-year-olds. A more recent study in adults found that warmer left-sided than right-sided temperatures were associated with increased impulsivity (active behavior), whereas a warmer right than left TMT was associated with cautious, passive behavior (Helton 2010). In short, studies on the association between TMT and behavior show somewhat inconsistent results. Age and type of sample may explain part of these inconsistencies.

Children who may be especially at risk for behavior and developmental problems are those who are adopted internationally, as they often lived under less than optimal conditions before the adoption (Hawk and McCall 2010; Juffer and Van IJzendoorn 2005). However, non-adopted Dutch children also present with a rather high prevalence of behavior problems: 21.6% had one or more Child Behavior Checklist (CBCL) syndrome scores above the borderline cut-off point and 12% had one or more syndrome scores above the clinical cut-off point (Koot 1993; Van Litsenburg et al. 2010). In general, it might thus not be expected that adopted children will show drastically higher numbers of behavior problems. It was indeed found that toddlers and preschoolers adopted from China into North America displayed fewer behavior problems than the normative sample, but they had relatively more sleep problems (Tan et al. 2007; Rettig and McCarthy-Rettig 2006). Sleep problems have in turn been associated with emotional and behavior problems in later childhood (Hemmi et al. 2011), but the mechanisms of this association are unclear (Gregory and O’Connor 2002).

Adopted children share the experience of being separated from their biological parents. During the pre-adoption period children are reared in either foster care or institutions. Foster care may provide children with a more stimulating environment and more individualized care than institutions (Gunnar et al. 2000; Van den Dries et al. 2010; Hawk and McCall 2010). The quality of the pre-adoption living arrangements may become especially important around 6 months of age; a sensitive period of attachment development. The development of the “clear-cut” attachment phase between 6 and 9 months of age is a sensitive period during which attachment to the primary caregiver develops (Bakermans-Kranenburg et al., 2011; Travers 2006). In agreement, Zeanah et al. (2011) concluded that long-term adverse effects of institutionalization have a greater chance to occur when children are institutionalized after the age of 6 months.

A large percentage of adopted children in the Netherlands have been adopted from China: in 2010, 44% of adopted children were born in China (Ministerie van Veiligheid en Justitie 2014). Due to this percentage and because previous research has largely focused on adoptees from other countries, we focused on Chinese adoptees in the current study.

In the present study we explored the relation between pre-adoption living arrangements at 6 to 9 months of age and TMT, and secondly, explored the relation between TMT and behavior problems in a sample of toddlers who were adopted from China. We hypothesized that institutional living conditions would predict a relatively higher left than right TMT due to the quality of care which is generally provided (Verhulst et al. 1992), and that higher left than right TMT would be associated with increased behavior problems, based on the only other previous TMT study that included the CBCL (Boyce et al. 1996). As relatively more sleep problems have been found in young children adopted from China (Tan et al. 2007), we explored the association between sleep problems and TMT asymmetry more specifically.

## Method

### Participants and procedure

Dutch families who adopted an infant from China (age 11–16 months at arrival in the Netherlands) were contacted through Dutch adoption agencies. As most infants adopted from China were female (Ministry of Justice 2009) due to the one-child policy in China (Johnson et al. 1998), only infants girls were included in our study. Written informed parental consent for participation of parents and their adopted children was obtained from all participants. The study was approved by the ethics committee of the Leiden University Medical Center, and was conducted in compliance with the Helsinki Declaration. One hundred families agreed to participate, of which eight dropped out due to personal reasons. Ninety (98%) of the remaining families were two-parent families. Thirteen families had previously adopted a child, and 13 families had one or more biological children. Most parents were highly educated (*M* =3.94, *SD* =0.72 on a scale ranging from: 1 = elementary school, to 5 = university degree). At arrival in the Netherlands, children were on average 13.03 months old (*SD* =1.35, range: 10.84 to 16.53).

Participating families were visited twice at home and came to the university for two lab visits. The first home and lab visits, hereafter called Time 1, took place two months after the child’s arrival. The mean age of the children at the first home visit was 15.24 months (*SD* =1.35), and the mean age at the first lab visit was 15.66 months (*SD* =1.42). The second home and lab visits, hereafter called Time 2, took place six months after arrival. The mean age at the second home visit was 19.33 months (*SD* =1.40) and 19.85 months (*SD* =1.48) at the lab visit. Visits were conducted with the child and the primary caregiver (90 mothers and 2 fathers).

### Measures

#### Pre-adoption living arrangements

The adoptive parents completed questionnaires on the background (e.g. “Has your daughter been placed in foster care in China? ) and living conditions (e.g. “How would you rate the institution on the following aspects: physical care, social-emotional care, presence of toys, hygiene, and overall atmosphere?”) during the pre-adoption period. Based on these responses, children were classified as either institutionalized (*n* =57) or having lived in foster care (*n* =34) between 6 and 9 months of age (Van den Dries et al. 2010).

#### Tympanic membrane temperatures

TMTs of the children were measured during the lab visits by the primary caregiver using the Braun Thermo Scan Pro 4000 digital thermometer as instructed by the researcher. The right side was measured first, followed by the left side, after which right and left-sided measurements were repeated. TMTs of some children could not be measured accurately resulting in 23 missing values at Time 1 and 16 missing values at Time 2.

The correlations between the first and second unilateral measurements of TMT at Time 1 (*N* =69) and Time 2 (*N* =76) ranged between *r* = .62 and *r* = .77; the means of the two measurements were used for analyses. TMT asymmetry was calculated by subtracting the mean left temperature from the mean right temperature. Consequently, a positive R-TMT asymmetry score represents a higher right- than left-sided TMT. Approximately 50% of participants had a higher right than left TMT at both Time 1 and Time 2. The correlation between TMT asymmetry at Time 1 and Time 2 for the total sample was *r* (67) = .24 (*p* = .06). Separate analyses revealed that TMT asymmetry was stable over time for institutionalized children (*r* (37) = .36, *p* = .02), but not for foster care children (*r* (23) = .11, *p* = .61), although these stabilities were not significantly different (*p* = .32). TMT means, standard deviations, and correlations for the total sample and institutionalized and foster care children separately are presented in Table 1. TMT asymmetry scores reversed in opposite directions for both groups between Time 1 and Time 2, but these differences were not significant for foster care children, *t*(24) = −1.82, *p* = .08, or institutionalized children, *t*(38) =1.95, *p* = .06.

**Table 1:** **Tympanic membrane temperatures and stability between two (Time 1) and six (Time 2) months after adoption**

	**Time 1** ***M*** **(** ***SD*** **)**	**Time 2** ***M*** **(** ***SD*** **)**	**Correlations (** ***r*** **) between Time 1 and Time 2**
Unilateral			
Measurements right TMT	37.07 (0.35)	37.03 (0.42)	.50 (*p* < .01)
Measurements left TMT	37.03 (0.34)	37.02 (0.34)	.41 (*p* < .01)
Bilateral			
R-TMT asymmetry			
Total sample	0.04 (0.36)	0.01 (0.34)	.24 (*p* = .06)
Institutionalized children	0.12 (0.36)	−0.05 (0.34)	.36 (*p* = .02)
Children in foster care	−0.08 (0.34)	0.09 (0.29)	.11 (*p* = .61)

#### Behavior problems

Child behavior problems were measured with the CBCL for children aged 1 to 5 years (Achenbach and Rescorla 2000) The caregivers indicated whether their child had displayed any of the 100 listed behaviors within the past two months on a 3-point scale (0 = not true, 1 = sometimes true, 2 = often or very true) prior to the home visits at Time 1 and Time 2. CBCL broadband scales were constructed as recommended for the Dutch version of the CBCL (Koot et al. 1997). The following five scales were composed: externalizing behavior problems (oppositional, aggressive, and overactive behaviors, e.g. “Cruel to animals”), internalizing behavior problems (withdrawn-depressed and anxious behaviors, e.g. “Clings to adults or too dependent”), sleep problems (e.g. difficulty going to bed, falling asleep, and sleeping through the night), somatic problems (e.g. Nausea, feels sick (without a medical cause)), and total behavior problems consisting of all previous named scales. Internal consistencies of the scales are presented in Table 2. The somatic scale had low internal consistency (α < .50) and was therefore excluded from further analyses.

**Table 2:** **Child Behavior Checklist (CBCL) scores two (Time 1) and six (Time 2) months after adoption**

	**Time 1**			**Time 2**		
**Scale**	**Internal consistency (α)**	**Institution** ***M*** **(** ***SD*** **)**	**Foster care** ***M*** **(** ***SD*** **)**	**Internal consistency (α)**	**Institution** ***M*** **(** ***SD*** **)**	**Foster care** ***M*** **(** ***SD*** **)**
Externalizing	.86	10.79 (6.80)	12.71 (6.81)	.90	9.66 (6.44)	13.99 (9.27)
Internalizing	.66	3.75 (2.88)	3.68 (2.93)	.72	2.90 (2.81)	2.79 (2.93)
Sleep	.74	2.82 (2.57)	3.21 (2.69)	.75	1.67 (2.30)	2.66 (2.45)
Somatic	.35	0.09 (0.34)	0.03 (0.17)	-.05	0.04 (0.19)	0.09 (0.29)
Total	.88	17.46 (10.13)	19.62 (10.05)	.91	14.38 (9.45)	19.52 (12.35)

### Data analysis

Bivariate associations between CBCL scales and TMT were examined in order to select relevant predictors to include in the model. Structural equation modeling using EQS 6.2 software for Windows (Bentler 2005) was employed to test the models predicting behavior problems and TMT asymmetry by pre-adoption experiences. Missing data was approached with the maximum likelihood method. Goodness of fit was assessed using three indices: a non-significant chi-square with a chi-square to degrees of freedom ratio (χ^2^/df) smaller than 2, a Comparative Fit Index (CFI) above .95, and a Root Mean-Square Error of Approximation (RMSEA) smaller than 0.06 (Hu and Bentler 1999).

## Results

### Descriptive statistics

Pre-adoption living arrangements in China were known for 91 participants (99%). More children were institutionalized (*n* =57) at this time than in foster care (*n* =34). These groups did not significantly differ on prematurity (*p* = .57), age at arrival in the Netherlands (*p* = .60), health at arrival (*p* = .49), age at first or second home visit (*p* = .74, *p* = .57) and lab visit (*p* = .66, *p* = .90), or head circumference (*p* = .93, *p* = .39), length (*p* = .64, *p* = .74) or weight (*p* = .52, *p* = .60) at Time 1 and Time 2.

R-TMT asymmetry scores at Time 1 were significantly less for children who had lived in foster care compared with institutionalized children, *t*(66) = −2.30, *p* = .03, *d* = −0.57. TMT asymmetry at Time 2 was not significantly different between these groups, *t*(73) =1.71, *p* = .09, *d* =0.40.

Scores of the CBCL subscales were stable over time (*r*’s > .50). Descriptive statistics are presented in Table 2. At Time 2, foster care children had significantly more externalizing behavior problems (*t*(89) = −2.62, *p* = .01) and total behavior problems (*t*(89) = −2.23, *p* = .03) compared with institutionalized children. Pearson correlations of CBCL scores of the total samples and institutionalized and foster care children with TMTs are presented in Table 3. Both the sleep problems scale (*r* = −.32, *p* < .01) and the total behavior problems scale (*r* = −.25, *p* = .04) were significantly associated with TMT asymmetry for the total sample at Time 1. However, the total behavior problems scale was only significantly associated with TMT asymmetry when the sleep problems scale was included; when this scale was removed the correlation was non-significant (*r* = −.19, *p* = .11). Furthermore, as the sleep problems scale was most strongly associated with the TMT measures for the total sample (i.e., left and bilateral TMT at Time 1) and a specifically higher prevalence of sleep problems had previously been found in a sample of Chinese adoptees (Tan et al. 2007; Rettig and McCarthy-Rettig 2006), this scale was selected for inclusion in the structural model. Sleep problems were not significantly different for children who had been institutionalized compared with foster care children at Time 1, *t*(89) = −0.67, *p* = .50, *d* = −0.14, or at Time 2, *t*(89) = −1.94, *p* = .06, *d* = −0.41. As the distribution of sleep problems scores was skewed at Time 2, the robust estimation approach was applied to assess the model.

**Table 3:** **Correlations between Child Behavior Checklist (CBCL) and tmt scores at two (Time 1) and six (Time 2) months after adoption**

	**Total sample (** ***N*** **=92)**			**Institutionalized children (** ***n*** **=57)**			**Children in foster care (** ***n*** **=34)**		
**Scale**	**R-Asymmetry**	**Right**	**Left**	**R-Asymmetry**	**Right**	**Left**	**R-Asymmetry**	**Right**	**Left**
Time 1									
Externalizing	-.23	-.11	.13	-.14	-.15	.02	-.26	-.02	.24
Internalizing	-.01	.05	.06	.21	-.06	-.27	-.16	.18	.39*
Sleep	-.32**	-.05	.30*	-.30	-.04	.28	-.25	.01	.28
Total	-.25*	-.08	.18	-.13	-.14	.02	-.28	.04	.35
Time 2									
Externalizing	.14	-.03	-.18	.05	-.03	-.10	.21	-.04	-.20
Internalizing	.15	.06	-.08	.20	.12	-.06	.10	-.01	-.09
Sleep	-.01	-.08	-.09	-.01	.06	.10	-.11	-.31	-.22
Total	.14	-.02	-.17	.09	.03	-.06	.16	-.09	-.21

*p < .05, **p < .01.

### Model assessment

First, it was hypothesized that pre-adoption living arrangements (Time 0) would directly predict R-TMT asymmetry at Time 1. A direct path between pre-adoption living arrangements and sleep problems at Time 1 was also included. Secondly, sleep problems and R-TMT asymmetry at Time 1 were expected to predict sleep problems and R-TMT at Time 2. Third, correlations between sleep problems and R-TMT at Time 1 and Time 2 were included as previous literature did not provide sufficient support to include a unidirectional path. Finally, cross-time, cross-construct paths between sleep problems at Time 1 and R-TMT at Time 2, and R-TMT at Time 1 and sleep problems at Time 2 were included.

**Figure 1:**
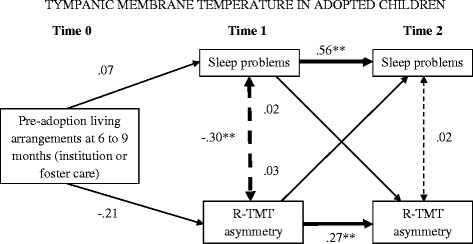
**Relations between pre-adoption arrangements, sleep problems and TMT asymmetry: hypothesized model with standardized β weights.** Pre-adoption living arrangements: 0 = institution, 1 = foster care. **p < .01.

This model did not provide an adequate fit to the data (χ^2^ [2, *N* =92] =8.26, *p* = .02, χ^2^/df =4.13; CFI = .92; RMSEA =0.17). R-TMT asymmetry (*β* = −.21, *p* = .42) and sleep problems (*β* = .07, *p* = .68) at Time 1 did not significantly differ for institutionalized compared with foster care children. The cross-time paths of sleep problems (*β* = .56, *p* < .001) and R-TMT asymmetry (*β* = .27, *p* < .01) were significant, indicating stability over time. The correlation between sleep problems and R-TMT was significant at Time 1 (*r* = −.30, *p* < .01), but not at Time 2 (*r* = .02, *p* = .42). The cross-time, cross-construct paths were not significant for sleep problems (*β* = .02, *p* = .38) or TMT asymmetry (*β* = .03, *p* = .41). Figure 1 presents the standardized parameter estimates for all hypothesized paths.

The Lagrange Multiplier test and Wald test were applied to modify the initial model in order to achieve a better fit (Tabachnick and Fidell 2007). Based on these tests, the cross-time paths for sleep problems and R-TMT, and the cross-construct correlation between sleep problems and R-TMT at Time 1 were retained. A direct path from pre-adoption living arrangements to R-TMT at Time 2 was added to the model. The fit of this adjusted model was satisfactory (χ^2^ [6, *N* =92] =8.40, *p* = .21, χ^2^/df =1.40; CFI = .98; RMSEA =0.05). The correlation between sleep problems at Time 1 and TMT asymmetry at Time 1 was significant (*r* = −33, *p* < .01). Sleep problems at Time 1 significantly predicted sleep problems at Time 2 (*β* = .55, *p* < .001) and explained 31% of the variance. Seventeen percent of the variance in R-TMT asymmetry at Time 2 was explained by pre-adoption living arrangements (*β* = .25, *p* < .01) and R-TMT asymmetry (*β* = .33, *p* < .001) at Time 1.

## Discussion

The current study is the first to explore the relation between TMT and behavior in a sample of adopted children. Findings suggest that for a sample of 92 infant girls adopted from China, TMT asymmetry was moderately stable across a time period of four months. In addition, pre-adoption living arrangements significantly predicted TMT asymmetry six months after the adoption and there was a significant association between TMT and sleep problems two months after adoption.

Structural equation modeling revealed that children who were institutionalized at 6 to 9 months of age had higher left than right TMTs at Time 2 compared with children who had been placed in foster care. In contrast, preliminary analyses indicated significantly higher right TMTs for institutionalized children at Time 1. However, living arrangements did not significantly predict R-TMT asymmetry at Time 1 when other paths were included. A possible explanation may be that TMT at Time 1 is more dependent on current environmental influences (state), whereas TMT at Time 2 reflects more stabilized (trait) differences and long-term effects of previous experiences, similarly to the development of attachment as illustrated by Dozier, Stovall, Albis, and Bater (2001), who measured attachment of children placed in foster care at least three months after placement to ensure measurement of consolidated (stabilized) attachment between child and caregiver. The same might be true of behavior problems: measurements at Time 1 require the caregiver to describe the behavior of the adopted child during the past two months, which are also the first two months after arrival during which many changes occur.

A higher left relative to right TMT was associated with increased sleep problems at Time 1 but not at Time 2. Our finding of an association between sleep problems and TMT asymmetry seems congruent with specifically higher prevalence of sleep problems in a sample of Chinese adoptees (Tan et al. 2007; Rettig and McCarthy-Rettig 2006). A possible explanation for this discontinuity is that children have adjusted to their adoptive families and their new living environments at Time 2 six months after adoption. In the first two months after placement in the adoptive family (Time 1) behavior may not yet have stabilized (Miller 2005; Dozier et al. 2001). In addition, correlational analyses indicated that a higher left TMT was associated with increased total behavior problems for the total sample at Time 1. However, this association was due to the inclusion of the sleep problems subscale in total behavior problems.

The direction of the relationship between TMT and behavior is consistent with a previous study that included the CBCL (Boyce et al. 1996) but contradicts some later findings of a positive correlation between right TMT and behavior problems (Boyce et al. 2002; Gunnar and Donzella 2004). We also found fewer associations between TMT and behavior problems than previous studies (e.g. Boyce et al. 1996), which may be explained by our specific (adoption) sample which precludes common genetic make-up associated with both TMT and parental perception of behavior problems. An alternative explanation may reside in the age of our participants, which was considerably younger than in previous studies examining the association between TMT and problem behavior. Our study is the first to include children before their second birthday, and as such may help to unravel developmental issues related to TMT.

Pre-adoption living arrangements were examined to provide more insight into environmental influences on TMT. Differences in TMT asymmetry between institutionalized and foster care children are consistent with previous findings. In another study, children who had been institutionalized had significantly smaller EEG alpha power than children who had been placed in foster care (Vanderwert et al. 2010), and had significantly smaller cortical gray and white matter volume and smaller posterior corpus callosum volume, whereas placement in foster care was associated with an increase in white matter (Sheridan et al. 2012). Our results similarly indicate that the quality of pre-adoption living arrangements is associated with neural development.

The present study has some limitations. First, single measurements of TMT were selected as opposed to continuous measurements. Future research should include long-term and continuous TMT ambulatory measurements, which may increase the validity of this measure and allows further exploration of TMT stability. Second, categorization of pre-adoption living arrangements was based on retrospective information, which is difficult to verify. Third, the finding that TMT asymmetry scores reverse in both groups over time, bordering on statistical significance, may complicate the relation between TMT and problem behavior. Fourth, the accuracy of infrared ear thermometers has been debated (Paes et al. 2010) and research is necessary to determine the accuracy of infrared ear thermometers in young children. Fifth, CBCL scores in our sample were low relative to Dutch and American samples on most scales except for the sleep problems scale. Furthermore, the current study is an exploratory investigation of the relation between pre-adoption living arrangements, TMT, and behavior problems in adopted toddlers. The nature of our study did not allow us to unravel the mechanisms underlying the association between TMT and behavior. More research, specifically including EEG, is necessary to examine the nature and mechanisms of these associations. Last, this study adds to a small body of empirical work on TMT in relation to behavior. TMT seems a very attractive easy-to-use assessment indicating lateralization and we found a significant and moderately strong association between sleep problems and TMT at Time 1. However, before we can conclude whether this is true and in what direction the associations between TMT and behavior problems point, a series of studies on various populations would be necessary. In addition, as a single study will not be sufficient to support or contradict the association between TMT and behavior, meta-analysis is required to draw more decisive conclusions on the relation between TMT and behavioral outcomes.

## Conclusions

Our findings with regard to pre-adoption living arrangements, TMT asymmetry, and sleep problems suggest that TMT is sensitive to early environmental influences and, as can be derived from previous studies, may be a biological marker of cerebral activation and vulnerability to the development of sleep problems in adopted infants. Although the underlying mechanisms of these associations require further exploration, our study may stimulate further work on the potential of TMT to serve as an indicator of behavior problems in children from adverse backgrounds.
